# Establishing Relationship between Vitamins, Total Phenolic and Total Flavonoid Content and Antioxidant Activities in Various Honey Types

**DOI:** 10.3390/molecules26154399

**Published:** 2021-07-21

**Authors:** Norhasnida Zawawi, Pei Juin Chong, Nurul Nadhirah Mohd Tom, Nurkhairina Solehah Saiful Anuar, Salma Malihah Mohammad, Norra Ismail, Arif Zaidi Jusoh

**Affiliations:** 1 Department of Food Science, Faculty of Food Science and Technology, Universiti Putra Malaysia, Serdang 43400, Selangor, Malaysia; peijuin0725@gmail.com (P.J.C.); nurulnadhirahmohdtom@gmail.com (N.N.M.T.); khairinasworks@gmail.com (N.S.S.A.); salmamalihah94@gmail.com (S.M.M.); 2 Laboratory of Halal Science Research, Halal Products Research Institute, Universiti Putra Malaysia, Serdang 43400, Selangor, Malaysia; 3 Food Science and Technology Research Center, Malaysian Agricultural Research and Development Institute (MARDI), Persiaran MARDI-UPM, Serdang 43400, Selangor, Malaysia; norra@mardi.gov.my (N.I.); arifzj@mardi.gov.my (A.Z.J.)

**Keywords:** honey, antioxidant activities, vitamin, total phenolic content, flavonoid content

## Abstract

Honey is a well-known natural sweetener and is rich in natural antioxidants that prevent the occurrence of oxidative stress, which is responsible for many human diseases. Some of the biochemical compounds in honey that contribute to this property are vitamins and phenolic compounds such as phenolic acids and flavonoids. However, the extent to which these molecules contribute towards the antioxidant capacity in vitro is inconsistently reported, especially with the different analytical methods used, as well as other extrinsic factors that influence these molecules’ availability. Therefore, by reviewing recently published works correlating the vitamin, total phenolic, and flavonoid content in honey with its antioxidant activities in vitro, this paper will establish a relationship between these parameters. Based on the literature, vitamins do not contribute to honey’s antioxidant capacity; however, the content of phenolic acids and flavonoids has an impact on honey’s antioxidant activity.

## 1. Introduction

Honey is a natural sweetener that is well-known all around the world. Naturally, it contains a concentrated sugar solution which is mostly fructose and glucose, making it favorable as a substitute for table sugar. It also contains different minor compounds, including polyphenols, enzymes, organic acids, and water-soluble vitamins [1], which contribute to its wide range of biological effects [2]. One of its sought after properties is its ability to counter oxidative stress, thereby acting as a potent antioxidant source.

Oxidative stress causes various pathological conditions such as cancer, neurological disorders, hypertension, and diabetes [3]. However, with a substantial amount of antioxidants the damage can be prevented. An antioxidant is any material that delays or stops the oxidation process of an oxidizable substance [4]. They include both enzymatic (superoxide dismutase, catalase etc.) and non-enzymatic molecules, which are the subject of interest in this review. An example of the important antioxidant compounds in honey are the phenolic compounds such as phenolic acid, flavonoids, and also vitamins [5,6,7].

There are various types of tests that can be used to measure honey’s antioxidant capacity in vitro, but none has been declared as the official method. Owing to some modifications within the same applied methods and test limitations, the results become difficult to compare. To counter this, researchers use more than one antioxidant assay and evaluate them using statistical analysis [8]. The measurement methods include ferric reducing antioxidant power (FRAP), inhibition of the 2,2′-azinobis-3-ethylbenozothiazoline-6-sulfonate (ABTS) radical cation, 1,1-diphenyl-2-picrylhydrazyl (DPPH), and many others [9]. Among these, the DPPH assay is most commonly applied to measure free radical scavenging activity due to its low cost [10].

Although honey has been reported to exhibit antioxidant properties, there is limited information about the specific cause of its antioxidant capacities [11]. Some studies have reported that the antioxidant properties are contributed by its different biochemical compounds found in varying degrees, and resulting from its floral origin, geographical origin, and environmental conditions [12].

This review focuses on analyzing the relationship between the vitamin, total phenolic, and total flavonoid contents with the antioxidant activities in honey in vitro. When these bioactive compounds increase, it is expected that honey’s antioxidant capacity will increase linearly.

## 2. Methodology

A literature search was conducted to identify recent articles and studies illustrating the relationship of antioxidant activities in honey to their vitamin, total phenolic, and flavonoid content. Several online databases were queried and used, including ScienceDirect, Wiley Online Library, PubMed, Scopus, and Web of Science. The keywords used individually and in combination as inclusion criteria for the articles to be taken into consideration for this review were honey, antioxidant, vitamin, phenolic acids, biochemical compounds, and total flavonoid. The inclusion criteria of the papers were (i) research that focused on the measurement of biochemical compound concentrations and antioxidant activities; (ii) papers written in English; and (iii) papers with accessible full text. This review covers a period of around 20 years, which includes publications from the year 2000 to 2020.

## 3. Vitamin and Antioxidant Activity

Vitamins are a large group of complex organic compounds that help to support body metabolism, growth, and development, and regulate the function of cells. To ensure a proper metabolic and cellular reaction in the body, vitamins are required in small amounts to work as important coenzymes and cofactors.

Vitamin A, vitamin C, and vitamin E are known to exhibit certain antioxidant properties. For instance, vitamin C’s antioxidant effects are due to its ability to reduce oxidation by reacting with superoxide ion O_2_^−^ and singlet oxygen such as HOO^−^ or OH^−^ through dehydrogenation to generate dehydroascorbate. Vitamin E can remove O_2_^−^ and quench singlet oxygen and superoxide dismutase by working glutathione peroxidase so that consumers have an antioxidant effect inside their body [13]. However, because of honey’s nature as an aqueous-based foodstuff [11], most studies only reported the content of water-soluble vitamins, such as Vitamin C and Vitamin B complex.

There are several methods for quantifying vitamins in honey, such as using a fluorometric method, titrimetric method, and chromatograpy [14]. The titrimetric method is widely employed for its simplicity and cost efficiency. However, when considering accuracy and precision, chromatography techniques such as high performance liquid chromatography (HPLC) are preferred [11].

Vitamin C and Vitamin B1, B2, and B3 were detected in honey samples of different botanical origins and geographical locations at various concentrations (Table 1). Manuka honey had the highest level of vitamins (1067.37 mg vitamin C/kg honey), followed by thyme honey (759 mg vitamin C/kg honey). The honey samples that had the lowest vitamin levels were eucalyptus 1 and multifloral honey from Province Leon, both having the same value at 3.40 mg vitamin C/kg honey.

There are numerous reports on vitamins in honey, but only a few studies have statistically analyzed the relationship between vitamin content and antioxidant activity. For example, Chua et al. [11] found that the water-soluble vitamin concentration in Gelam, Tualang, and Acacia honey in Malaysia was significantly correlated with FRAP values (*r* = 0.4338, *p* < 0.05). Although there was a high correlation with DPPH activity, it was insignificant (*r* = 0.8226, *p* > 0.05). Gelam had the highest vitamin content and the highest antioxidant capacity.

Moreover, Combarros-Fuertes and Azza et al. [15,16] found no significant relationship between vitamin content and DPPH activity in various honey samples. This suggests that vitamins, in small amounts, do not contribute towards the antioxidant activity of a sample. A higher vitamin content has been shown to reduce the β-carotene bleaching inhibition in honey (*r* = −0.61; *p* < 0.05), which is believed to be due to the formation of ascorbyl radical [15].

In addition, many studies did not correlate vitamin content with antioxidant activity [17,18,19,20,21,22,23,24,25,26,27,28] but the vitamin content was significantly different (*p* < 0.05) across each sample.

## 4. Total Phenolic Content and Antioxidant Activity

Phenolic compounds are one of the most important compounds contributing to the antioxidant activity of honey [5]. Based on carbon chain classification, there are 16 classes of phenolic compounds [31]. In honey, the two most common are phenolic acid (non-flovonoid) and flavonoids [32]. Phenolic content in honey varies among honey types and geographical origins.

The recovery of phenolic compounds is primarily affected by sample preparation and the method of extraction. Some important variables need to be taken into account such as type of solvent and the time and temperature parameters of the extraction process [33]. The most common method used to quantify total phenolic content are the Folin–Denis and Folin–Ciocalteu methods [33]. To identify and quantify individual compounds, chromatography approaches such as HPLC or liquid chromatography (LC coupled with UV-VIS or diode array detector) were adopted [20,34,35,36,37].

In Table 2, 16 different phenolic compounds detected in honey samples are shown. Overall, multifloral 1 honey from Brazil [34] had the highest phenolic content (78.2 ± 2.7 mg/g GAE), while the lowest was reported in Clover honey (0.65 ± 0.42 mg/g GAE) [35]. Reports have mostly shown a strong correlation between total phenolic content and the antioxidant capacity of honey samples (Table 1).

Lianda et al. [34] investigated Brazilian honey and found multifloral honey to have the highest TPC value (78.2 ± 2.7 mg/g GAE) and the lowest IC_50_ value (10.81 ± 0.50 mg/mL). IC_50_ corresponds to the sample concentration needed to scavenge 50% of DPPH· radicals. Therefore, low IC_50_ is equivalent to a high scavenging power. However, based on FRAP assay, orange blossom 1 honey had the highest FRAP value (438.69 ± 2.78 mol Fe(II)/100 g). Nevertheless, analyses showed a strong correlation between TPC and DPPH_IC50_ (*r* = −0.8918) and FRAP assay (*r* = 0.9258).

In a study by Can et al. [35], the total phenolic content of Turkish honey was strongly correlated with a FRAP assay (*r* = 0.81, *p* < 0.05). Chestnut and oak honey had the highest FRAP values, followed by heather, pine, and Jerusalem tea honey.

Similarly, Nascimento et al. [36] also found a strong correlation between TPC and DPPH_IC50_ activity (*r* = −0.7582) and FRAP assay (*r* = 0.8594) in Brazilian honey. In addition, they found abundant gallic acid, a phenolic compound with antioxidant properties in Japanese grape, mastic, and wildflower honey that influenced the FRAP values (*r* = 0.5202).

Khalil et al. [37] compared Malaysian honey with Manuka honey from New Zealand and discovered that TPC was highly correlated with DPPH % inhibition (*r* = 0.976, *p* < 0.01) and FRAP assay (*r* = 0.965, *p* < 0.01), showing Tualang honey as the richest in phenolic compounds and highest in radical scavenging activities. The strong correlation of TPC in both assays was also observed in all samples of Kosovo honey [38]. Honey samples from the forest had the highest TPC, DPPH (%), and FRAP values and the correlation with these two assays were (DPPH; *r* = 0.931, *p* < 0.01) and (FRAP; *r* = 0.878, *p* < 0.01).

However, not all studies showed a strong relationship with the assays. For example, Šarić et al. [39] only discovered a strong correlation in TPC of multifloral honey from Croatia with the FRAP value (*r* = 0.8325). While the relationship with DPPH_IC50_ was unfavorable (*r* = 0.5791) and insignificant. Ranneh [20] also found no significant correlation between TPC and DPPH and ABTS parameters in Tualang and Kelulut honey.

Despite this, most results suggest that phenolic compounds are partially responsible for honey’s antioxidant properties.

## 5. Flavonoid Content and Antioxidant Activity

Flavonoids are known to be polyphenolic compounds comprising two phenyl rings linked by a propane bridge, resulting in a characteristic 15-carbon (C6-C3-C6) flavan skeleton [40]. They can be regarded as a class of phenolic compounds having a low molecular weight and are widely distributed in the plant kingdom. In higher plants, they represent one of the most distinctive compound groups. In most angiosperm families, nearly all flavonoids are easily detected as flower pigments. Flavonoids can be found naturally in all parts of plants and were discovered to be the major colouring component of the flowering plants [41]. Anthocyanins, flavonols, flavan-3-ols, flavanones, flavones, and isoflavones are some of the main groups of flavonoids.

Honey can be distinguished by its composition, such as the presence of flavonoid compounds. In various plant species, flavonoids are the dominant class of secondary metabolites and occur in various tissues and organs [42]. Antioxidant properties are also controlled by the subgroup of flavonoid compounds found in honey. Each subgroup has different degrees of unsaturation and oxidation of the carbon ring, depending on the location of the C ring attached to the B-ring [43]. The flavonoid subgroups found in honey are mostly flavonols, flavanone, and flavones.

To estimate the flavonoid content in honey, the calorimetric method using aluminium chloride is widely applied [11,20,36,37,39,44,45]. There may be slight modifications from the original procedure but the underlying principle is the same. Flavonoid reacts with aluminium chloride to form a stable acid complex which is detected using spectrophotometer [46]. Then, flavonoids are individually identified and quantified using a chromatographic approach, such as UPLC [11], HPLC [36,37,44], or LC [20].

Table 3 shows the flavonoid content with antioxidant activities in various types of honey. The most common flavonoids detected from different honey samples were gallic acid and caffeic acid. Similarly to TPC, the relationship between TFC and the antioxidant assays mostly showed a moderate to strong correlation. For example, based on DPPH % radical scavenging activity (RSA), there was strong relationship between TFC and DPPH (% RSA). Meanwhile, the lowest (*r* = 0.888, *p* < 0.001) was recorded by Khalil et al. [37].

Sousa et al. [47] found a stronger relationship between these two variables in Brazilian honey (*r* = 0.9377, *p* < 0.01). Monofloral honey from Jandaira, Brazil, had the highest RSA (46.9 ± 1.9% RSA), with a TFC content of 4.2 ± 0.6 mg GAE/100 g.

A-Farsi [45] investigated Omani honey and found a TFC relationship with DPPH_IC50_ at *r* = −0.616. However, Saric et al. [39] observed contradictory results when using two different assays. The correlation between TFC and FRAP assays was higher (*r* = 0.7062), signaling a flavonoid compound attribution towards the FRAP analysis. However, when evaluating TFC and DPPH_IC50_, the relationship was positive, with a relationship coefficient of 0.4272.

Nascimento et al. [36] stated that there was a relationship between TFC and DPPH_IC50_, without stating whether it was positive or negative. However, the authors reported a strong relationship between TFC and FRAP values (*r* = 0.8435, *p* < 0.005), with Mastic honey being quantified with the highest values of both of these variables.

In the case of Ranneh et al. [20], they observed a significant relationship between TFC of Kelulut (*Trigona*) and Tualang (*Apis dorsata*) honey with DPPH % (*r* = 0.922 and *r* = 0.936, respectively, *p* < 0.01), but not with ABTS assay. Kelulut honey had the highest TFC, with 101.5 ± 11.4 mgCE/kg. Overall, the authors concluded that Kelulut honey has a stronger antioxidant capacity than Tualang honey.

## 6. Conclusions

This review paper has established correlations between vitamin, total phenolic, and flavonoid contents with the antioxidant activities in vitro. Data on the relationship between vitamins and antioxidant activities are scarce, but based on the available reports, vitamins do not contribute to honey’s antioxidant capacity. Although vitamins are well-known antioxidant molecules, their presence in minute amounts could not provide a substantial contribution towards honey’s antioxidant activity. On the other hand, total phenolic and flavonoid contents are associated with antioxidant activities in vitro. The phenolic compounds are influenced by several factors, including geographical location, botanical origin, type of phenolic compounds, storage duration, and processing method. In addition, because there is no standardized method for measuring honey’s antioxidant activity, comparisons between studies are difficult, especially when the expressed units are different (i.e., TPC content and FRAP values). Thus, it is recommended to establish a standard to measure honey’s antioxidant capacity, in order to obtain reliable data that can be compared across various studies. This review focused on in vitro antioxidant studies. Naturally, there are limitations to these results, as it does not consider the physiological parameters that can be observed in in vivo settings. The bioavailability and the synergistic/antagonistic effect between these compounds in humans are still vague. However, the strong correlation seen in this review provides fundamental information that phenolic compounds in honey do have positive effects on antioxidant activity. The in vitro studies are increasing rapidly and the in vivo studies are also catching up. More clinical research should instead be done to validate honey as an alternative medicine based on its antioxidant potential.

## Figures and Tables

**Table 1:** Vitamins concentrations and antioxidant activities of honey.

Botanical Origin	Bee Species	Vitamin	Quantification Method	Vitamin Concentration (mg/kg)	DPPH		FRAP	CBI (%)	ABTS	Relationship	References
					IC_50_ (mg/mL)	% Inhibition					
Gelam 1 (*Melaleuca cajuputi*)	*Apis* *dorsata*	Vitamin B1	RP-HPLC with PDA detector	13.85	15.681	N/A	82.529 mg TE/100g honey	67.41	N/A	Vitamin with DPPH: *r* = 0.8226, *p* > 0.05 Vitamin with FRAP: *r* = 0.4338, *p* < 0.05 Vitamin with CBI: *r* = 0.2649, *p* > 0.05	[11]
		Vitamin B3		355.38							
		Vitamin C		67.36							
Acacia 1 (*Robinia Pseudocacia*)	*Apis mellifera*	Vitamin B1		11.85	29.846		82.386 mg	74.66			
		Vitamin B3		134.67							
		Vitamin C		62.80							
Kedah 1 (Malaysia) (Tualang forest honey)	*Apis* *dorsata*	Vitamin B3		170.38	48.896		52.386 mg	35.81			
		Vitamin B3		52.20							
Avocado (*Persea americana*)	*Apis Mellifera*	Vitamin C	Titrimetric Method (AOAC 967.21)	59.50	13.8	N/A	N/A	56.9	N/A	Vitamin with DPPH: *r* = 0 Vitamin with CBI: *r* = − 0.61, *p* < 0.05	[15]
Chestnut 1 (*Castanea sativa*)				36.40	23.0			66.8			
Rosemary 1 (*Rosmarinus officinalis*)				45.10	202			28.3			
Eucalyptus 1 (*Eucalyptus* sp.)				3.40	202			71.8			
Thyme (*Thymus* sp.)				759.00	5.46			−1.34			
Province of Granada (Spanish)				91.10	9.25			32.9			
Province of Cuenca (Spanish)				24.10	38.0			58.4			
Province of Pontevedra (Spanish)				13.50	28.9			92.9			
Province of Leon (Spanish)				3.40	54.0			68.1			
Strawberry tree (*Arbutus unedo*)	*Apis mellifera/Apis cerana/Trigona minangkabau*	Vitamin C	RP-HPLC	71.57	N/A	N/A	N/A	N/A	0.44	Vitamin with ABTS *r* = N/A, *p* > 0.01	[16]
Cardoon (*Carlina racemos*)	*Apis florea/Apis mellifera*			93.43					0.85		
Carob *(Ceratonia siliqua*)	*Apis mellifera*			138.87					1.55		
Orange 2 (*citrus sinensis*)	*Apis mellifera/Apis cerana*			77.70					4.04		
Sunflower (*Helianthus annuus*)	*Apis mellifera/Apis dorsata/Apis cerana/Trigona laeviceps*			98.57					3.17		
Algarve (Portugal)	*Vespa Velutina*			95.73					1.73		
Sleman, Yogyakarta (Indonesia)	*Tetragonula laeviceps*	Vitamin C	Titrimetric Method	78.80	N/A	90.50	N/A	N/A	N/A	Vitamin with DPPH (%) *r* *=* 0.56, (not significant)	[29]
Klaten, Central Java (Indonesia)				65.10		91.20					
Nglipar, Yogyakarta (Indonesia)				56.70		47.3					
Manuka *(Leptospermum scoparium)*	*Apis mellifera/*	Vitamin C	Titrimetric Method	1067.37	N/A	N/A	434.3 μmol Fe(II)/100g honey	N/A	N/A	Vitamin with FRAP *r* = N/A	[30]
Longan *(Dimocarpus longan)*	*Apis cerana/Apis dorsata*			190.61			258.9 μmol				
Mangosteen *(Garcinia mangostana)*	*Apis mellifera*/*Apis cerana*			379.31			908.3 μmol				
Pararubber *(Hevea brasiliensis)*	*Apis mellifera*			185.82			262.2 μmol				

RP: reversed phase, HPLC: high performance liquid chromatography, PDA: photodiode array, N/A: not available, CBI: β-carotene bleaching inhibition.

**Table 2:** The phenolic content and antioxidant activities of honey.

Botanical Origin	Bee Species	Method	Phenolic Compounds	TPC (mg/100 g)	DPPH		FRAP	Relationship	Reference
					% Inhibition	IC_50_ (mg/mL)			
Orange Blossom 1	*Apis mellifera*	Folin–Dennis method with LC-DAD	Gallic acid, vanillic acid, syringic acid, quercetin.	35.7 ± 2.4	ND	36.22 ± 3.82	438.69 ± 2.78 (mol Fe(II)/100 g)	TPC with FRAP *r* = 0.857, *p* < 0.01 TPC with DPPH_IC50_ *r* = −0.8918, *p* < 0.01	[34]
Orange Blossom 2			protocatechuic acid, *p*-coumaric acid, syringic acid, cinnamic acid	38.8 ± 3.6		40.80 ± 4.68	376.66 ± 1.60		
Orange Blossom 3			protocatechuic acid	53.2 ± 2.9		29.85 ± 2.67	375.73 ± 6.99		
Orange Blossom 4			*p*-hydroxybenzoic acid, vanillic acid, *p*-coumaric acid	40.1 ± 2.9		33.21 ± 2.51	34.99 ± 4.24		
Orange Blossom 5			protocatechuic acid, syringic acid, *p*-coumaric acid, cinnamic acid	34.0 ± 7.58		52.64 ± 4.70	303.51 ± 1.60		
Multifloral 1 (Brazil)			gallic acid, vanillic acid, *p*-hydroxybenzoic acid, Sinapic acid, Morin cinnamic acid.	78.2 ± 2.7		10.81 ± 0.50	95.18 ± 3.21		
Multifloral 2 (Brazil)			protocatechuic acid, *p*-coumaric acid, cinnamic acid	42.8 ± 1.9		19.74 ± 1.62	408.14 ± 10.02		
Multifloral 3 (Brazil)			protocatechuic acid, quercetin, *p*-hydroxybenzoic acid	57.2 ± 2.4		18.42 ± 1.47	109.99 ± 11.23		
Multifloral 4 (Brazil)			vanillic acid, *p*-hydroxybenzoic acid, p-coumaric acid, morin	54.0 ± 2.3		17.52 ± 1.10	78.51 ± 4.24		
Chestnut	*Apis mellifera*	Folin–Ciocalteu method with HPLC-UV-VIS	Gallic acid, protocatechuic acid, *p*-hydroxybenzoic acid, vanillic acid, caffeic acid, *p*-coumaric acid, ferulic acid, quercetin.	8.10 ± 2.56	ND	20.05 ± 5.42	4.30 ± 0.13 (µmol FeSO_4_•7H_2_O/g)	TPC with FRAP *r* = 0.81, *p* < 0.05 TPC with DPPH *r* = N/A	[35]
Astragalus			Gallic acid, *p*-coumaric acid, *p*-hydroxybenzoic acid, vanillic acid, caffeic acid, protocatechuic acid, ferulic acid, rutin, apigenin	0.86 ± 0.49		123.56 ± 25.12	0.66 ± 0.74		
Heather			Gallic acid, protocatechuic acid, protocatechuic acid, catechin, caffeic acid, *p*-coumaric acid, aquercetin.	5.84 ± 1.80		1.42 ± 0.28	27.84 ± 13.20		
Clover			*p*-coumaric acid, *p*-hydroxybenzoic acid, caffeic acid	0.65 ± 0.42		98.19 ± 58.03	0.59 ± 0.21		
Lavender			*p*-hydroxybenzoic acid, catechin, caffeic acid, epicatechin *p*-coumaric acid, rutin.	2.20 ± 1.54		70.20 ± 31.50	0.67 ± 0.25		
Lime			protocatechuic acid, *p*-hydroxybenzoic acid, caffeic acid, apigenin.	0.95 ± 0.18		76.20 ± 12.30	0.86 ± 0.12		
Jerusalem Tea			*p*-hydroxybenzoic acid, vanillic acid, caffeic acid, *p*-coumaric acid, ferulic acid, quercetin, apigenin.	2.80 ± 1.10		61.05 ± 5.20	0.65 ± 0.46		
Common eryngo			*p*-hydroxybenzoic acid, catechin, caffeic acid, *p*-coumaric acid, ferulic acid.	0.85 ± 0.64		60.08 ± 6.10	2.27 ± 0.96		
Chaste tree			*p*-hydroxybenzoic acid, catechin, caffeic acid, *p*-coumaric acid, ferulic acid, apigenin.	0.95 ± 0.24		121.05 ± 20.40	0.67 ± 0.46		
Rhododendron			Gallic acid, protocatechuic acid, *p*-hydroxybenzoic acid, vanilic acid, caffeic acid, *p*-coumaric acid, ferulic acid, quercetin, apigenin, kaempferol.	0.92 ± 0.39		78.06 ± 28.65	0.67 ± 0.22		
Oak			Gallic acid, protocatechuic acid, *p*-hydroxybenzoic acid, caffeic acid, syringic acid, epicatechin, *p*-coumaric acid, ferulic acid, rutin	3.10 ± 0.56		12.56 ± 2.50	3.07 ± 0.84		
Pine			Protocatechuic acid, *p*-hydroxybenzoic acid, catechin, vanillic acid, caffeic acid, syringic acid, *p*-coumaric acid, ferulic acid, rutin, quercetin, kaempferol.	1.58 ± 1.30		44.30 ± 25.07	1.48 ± 0.83		
Acacia			*p*-hydroxybenzoic acid, caffeic acid, synringic acid, *p*-coumaric acid, ferulic acid, apigenin, kaempferol, isorhamnetin.	1.58 ± 0.22		152.40 ± 62.00	0.64 ± 0.34		
Japanese Grape	*Apis mellifera*	Folin–Ciocalteu method with RP-HPLC-UV-VIS	Gallic acid, *p*-coumaric acid, quercetin.	30.5 ± 14.4	ND	179.12 ± 101.8	0.33 ± 0.6 (μmolET.g−1)	TPC with FRAP *r* = 0.8594, *p* < 0.001 TPC with DPPH_IC50_ *r* = −0.7582, *p* < 0.001	[36]
Mastic			Gallic acid, cinnamic acid, quercetin.	63.5 ± 17.3		62.2 ± 29.7	1.8 ± 0.5		
Quitoco			Gallic acid, quercetin.	50.2 ± 17.7		180.0 ± 82.8	0.4 ± 0.3		
Wild Flower			Gallic acid.	56.5 ± 0.00		86.04 ± 0.00	0.97 ± 0.0		
Manuka (New Zealand)	*Apis mellifera*	Folin–Ciocalteu method with HPLC-DAD	Caffeic acid, *p*-coumaric acid, catechin.	52.63 ± 1.21	N/A	4.71 ± 0.36	1.295 ± 0.01 (μM Fe[II]/kg)	TPC with FRAP *r* = 0.965, *p* < 0.01 TPC with DPPH % *r* = 0.976, *p* < 0.01	[37]
Borneo Tropical (Malaysia)	*Apis cerana*		Catechin, caffeic acid.	15.21 ± 0.51		17.51 ± 0.51	0.492 ± 0.01		
Tualang (Malaysia)	*Apis dorsata*		Catechin, gallic acid, syringic acid, caffeic acid.	28.87 ± 0.41		8.60 ± 0.66	0.707 ± 0.007		
Multiflora (Bednja)	N/A	Folin–Ciocalteu method	N/A	19.581 ± 0.578	N/A	11.94 ± 0.71	398.02 ± 36.87 (mM of Fe(II) in 10% honey solution.	TPC with DPPH_IC50_: *r* = 0.5791, *p* = N/A TPC with FRAP: *r* = 0.8325, *p* = N/A	[39]
Multiflora (Ivanec)				20.876 ± 15.94		11.86 ± 1.99	283.98 ± 30.23		
Multiflora (Novi Marof)				23.638 ± 0.805		11.26 ± 1.06	351.10 ± 86.09		
Multiflora (Ludbreg)				16.362 ± 1.322		13.73 ± 6.95	244.55 ± 32.93		
Forest (Kosovo)	N/A	Folin–Ciocalteu method	N/A	84.17 ± 30.40	66.92 ± 23.18	N/A	22.39 ± 12.86 (mg TE/100 g)	TPC with DPPH % *r* = 0.804, *p* < 0.01 TPC with FRAP: *r* = 0.829, *p* = 0.01	[38]
Meadow				46.48 ± 16.59	42.23 ± 25.06		10.73 ± 7.46		
Mixed				46.33 ± 18.95	34.49 ± 22.50		7.76 ± 7.14		
Chestnut				35.77 ± 8.26	31.35 ± 9.44		8.50 ± 2.51		
Acacia				25.76 ± 10.16	22.23 ± 7.82		3.65 ± 1.96		
Lime				74.10	65.15		16.63		
Pine				28.06	38.70		9.75		
TH1 Multifloral (Malaysia)	*Apis dorsata*	Folin–Ciocalteu method with LC-MS/MS	Gallic acid, Caffeic acid, Chrysin, Cinnamic acid, Hydroxycinnamic acid, Kaempferol, *p*-coumaric acid	13.942 ± 1.37	N/A	ND	ND	TPC with DPPH % *r* = 0.584	[20]
TH2 Multifloral (Malaysia)			Gallic acid, Caffeic acid, Syringic acid, Catechine, Apigenin, Chrysin, Cinnamic acid, Hydroxycinnamic acid, quercetin-3-O-rutinosid	18.393 ± 2.41					
KH1 Unifloral (Malaysia)	*Trigona*	Folin–Ciocalteu method with LC-MS/MS	Gallic acid, Caffeic acid, Syringic acid, Apigenin, Chrysin, Cinnamic acid, Hydroxycinnamic acid, Kaempferol, *p*-coumaric acid, quercetin-3-O-rutinosid	22.809 ± 0.79	N/A	ND	ND	TPC with DPPH % *r* = 0.607	[20]
KH2 Multifloral (Malaysia)			Gallic acid, Caffeic acid, Caffeic acid, Syringic acid, Catechine, Apigenin, Chrysin, Cinnamic acid, Hydroxycinnamic acid, Kaempferol, *p*-coumaric acid, quercetin-3-O-rutinosid, Hydroxybenzoic acid	23.528 ± 0.06					

LC: liquid chromatography, RP: reversed phase, HPLC: high performance liquid chromatography, MS: mass spectroscopy, DAD: diode-array detection, UV-VIS: ultraviolet/visible light detector, N/A: not available, ND: not determined.

**Table 3:** The flavonoid compounds and antioxidant activities of honeys.

Botanical Origin	Bee Species	Method	Flavonoid (mg/g)	TFC	DPPH		FRAP	Relationship	Reference
					IC_50_ (mg/mL)	% Inhibition			
Eucalyptus	*Apis mellifera*	Colorimetric method with RP-HPLC-UV/VIS	Myricetin, quercetin	0.75 ± 0.5 (mg/g)	65.09 ± 35.5	N/D	1.34 ± 0.4 (μmol ET/g)	TFC with DPPH_IC50_: *r* = correlated, *p* < 0.05 TFC with FRAP: *r* = 0.8435, *p* < 0.05	[36]
Mastic			Quercetin	2.1 ± 1.1	62.2 ± 29.7		1.8 ± 0.5		
Japanese grape			Quercetin	0.2 ± 0.7	179.12 ± 101.8		0.33 ± 0.6		
Quitoco			Quercetin	0.0 ± 0.6	180.0 ± 82.8		0.4 ± 0.3		
Wild flower (Brazil)			N/D	N/D	0.40		86.04		
Polyfloral (Brazil)			Myricetin, quercetin	1.2 ± 0.7	82.6 ± 37.6		1.2 ± 0.5		
Juazeiro	*Meliponini* *subnitida*	Colorimetric method with HPLC-UV	Myricetin, quercetin, catechin, rutin, kaempferol, hesperetin, naringenin, chrysin	4.2 ± 0.6 (mg GAE/100 g)	N/D	46.9 ± 1.9	N/D	TFC with DPPH % *r* = 0.9377, *p* < 0.01	[44]
	*Meliponini* *scutellaris*			4.4 ± 0.2		29.5 ± 3.4			
Malicia	*Meliponini* *subnitida*			4.1 ± 0.4		23.3 ± 1.4			
	*Meliponini* *scutellaris*			4.0 ± 0.4		11.2 ± 1.3			
Velame Branco	*Meliponini* *subnitida*			2.6 ± 0.6		40.1 ± 3.2			
	*Meliponini* *scutellaris*			1.9 ± 0.1		27.5 ± 0.5			
Jurema Branca	*Meliponini* *subnitida*			2.4 ± 0.1		22.7 ± 1.6			
	*Meliponini* *scutellaris*			2.1 ± 0.5		24.6 ± 0.4			
Gelam (Malaysia)	*Apis dorsata*	Colorimetric method with HPLC-DAD	Catechin, naringenin, luteolin, kaempferol, apigenin.	0.02531 ± 0.00037 (mg CEQ/g)	14.36 ± 0.83	N/A	0.64428 ± 0.00953 (μM Fe(II)/g)	TFC with DPPH % *r* = 0.888, *p* < 0.01 TFC with FRAP: *r* = 0.899, *p* < 0.01	[37]
Manuka (Malaysia)	*Apis mellifera*		Catechin	0.03455 ± 0.00045	4.71 ± 0.36		1.29534 ± 0.01035		
Borneo tropical (Malaysia)	*Apis cerana*		Catechin	0.01152 ± 0.00027	17.51 ± 0.51		0.49204 ± 0.01125		
Tualang 2 (Malaysia)	*Apis dorsata*		Catechin, kaempferol	0.02052 ± 0.00021	8.60 ± 0.66		0.70691 ± 0.00728		
Tualang 3 (Malaysia)	*Apis dorsata*		Catechin, Kaempferol	0.02173 ± 0.00043	6.94 ± 0.08		0.65173 ± 0.0088		
Tualang 4 (Malaysia)	*Apis dorsata*		Catechin, naringenin	0.02531 ± 0.00037	5.24 ± 0.40		0.89215 ± 0.00497 (		
Multifloral 1 (Omani)	*Apis mellifera*	Colorimetric method	N/D	0.925 (mg/g)	144.5 (mg/mL)	N/A	N/D	TFC with DPPH: *r* = −0.616	[45]
Tualang (Malaysia)	*Apis dorsata*	Colorimetric method with UPLC-MS/MS	Pinobanksin-3-O-propionate, Pinobanksin-3-O-butyrate, Quercetin	18.511 ± 2.803 (mg/g)	48.896	N/A	52.386 ± 5.192 (mg TE/100 g)	TFC with DPPH % *r* = 0.9276, *p* > 0.05 TFC with FRAP: *r* = 0.991, *p* < 0.05	[11]
Gelam (Malaysia)				32.886 ± 0.780	15.681		82.529± 5.032		
Acacia (Malaysia)				30.741 ± 2.886	29.846		82.386 ± 5.930		
Multiflora (Bednja, Croatia)	N/D	Colorimetric method	N/D	28.05 ± 0.47 (mg/g)	11.94 ± 0.71	N/A	398.02 ± 36.87 (mM of Fe(II) in 10% honey solution.	TFC with DPPH_IC50_: *r* = 0.4272 TFC with FRAP: *r* = 0.7062	[39]
TH1 Multifloral (Malaysia)	*Apis dorsata*	Colorimetric method with LC-ESI-MS/MS	Chrysin, Kaempferol,	64.72 ± 11.4 (mg CE/kg)	N/A	ND	ND	TFC with DPPH % *r* = 0.922, *p* < 0.01	[20]
TH2 Multifloral (Malaysia)			Catechin, Apigenin, Chrysin, quercetin-3-O-rutinosid	18.393 ± 2.41					
KH1 Unifloral (Malaysia)	*Trigona*		Apigenin, Chrysin, Kaempferol, quercetin-3-O-rutinosid	22.809 ± 0.79 (mg CE/kg)				TFC with DPPH % *r* = 0.936, *p* < 0.01	
KH2 Multifloral (Malaysia)			Catechin, Apigenin, Chrysin, Kaempferol, quercetin-3-O-rutinosid,	23.528 ± 0.06 (mg CE/kg)					

RP: reversed phase, HPLC: high performance liquid chromatography, UV-VIS: ultraviolet/visible light detector, DAD: diode-array detection, UPLC; ultra-performance liquid chromatography, MS: mass spectroscopy, LC: liquid chromatography, ESI: electrospray ionization.

## References

[1] Abou-Shaara H. (2015). The origin of honey bees, life: A viewpoint. Artic. J. Entomol. Zool. Stud..

[2] Pasupuleti V.R., Sammugam L., Ramesh N., Gan S.H. (2017). Honey, Propolis, and Royal Jelly: A Comprehensive Review of Their Biological Actions and Health Benefits. Oxid. Med. Cell. Longev..

[3] Birben E., Sahiner U.M., Sackesen C., Erzurum S., Kalayci O. (2012). Oxidative stress and antioxidant defense. World Allergy Organ. J..

[4] James O.O., Mesubi M.A., Usman L.A., Yeye S.O., Ajanaku K.O., Ogunniran K.O., Ajani O.O., Siyanbola T.O. (2009). Physical characterisation of some honey samples from North-central Nigeria. Int. J. Phys. Sci..

[5] Cheung Y., Meenu M., Yu X., Xu B. (2019). Phenolic acids and flavonoids profiles of commercial honey from different floral sources and geographic sources. Int. J. Food Prop..

[6] Khalil M.I., Sulaiman S.A., Boukraa L. (2010). Antioxidant Properties of Honey and Its Role in Preventing Health Disorder. Open Nutraceuticals J..

[7] Nayik G.A., Suhag Y., Majid I., Nanda V. (2018). Discrimination of high altitude Indian honey by chemometric approach according to their antioxidant properties and macro minerals. J. Saudi Soc. Agric. Sci..

[8] Maurya S., Kushwaha A.K., Singh S., Singh G. (2014). An overview on antioxidative potential of honey from different flora and geographical origins. Indian J. Nat. Prod. Resour..

[9] Beretta G., Granata P., Ferrero M., Orioli M., Facino R.M. (2004). Standardization of antioxidant properties of honey by a combination of spectrophotometric/fluorimetric assays and chemometrics. Anal. Chim. Acta..

[10] Ahn M.R., Kumazawa S., Usui Y., Nakamura J., Matsuka M., Zhu F., Nakayama T. (2007). Antioxidant activity and constituents of propolis collected in various areas of China. Food Chem..

[11] Chua L.S., Rahaman N.L.A., Adnan N.A., Eddie Tan T.T. (2013). Antioxidant activity of three honey samples in relation with their biochemical components. J. Anal. Methods Chem..

[12] Baroni M.V., Podio N.S., Badini R.G., Inga M., Ostera H.A., Cagnoni M., Gautier E.A., García P.P., Hoogewerff J., Wunderlin D.A. (2015). Linking soil, water, and honey composition to assess the geographical origin of Argentinean honey by multielemental and isotopic analyses. J. Agric. Food Chem..

[13] Li S., Chen G., Zhang C., Wu M., Wu S., Liu Q. (2010). Research progress of natural antioxidants in foods for the treatment of diseases. Food Sci. Hum. Wellness..

[14] Nielsen S.S. (2010). Vitamin Analysis. Food Analysis.

[15] Combarros-Fuertes P., Estevinho L.M., Dias L.G., Castro J.M., Tomás-Barberán F.A., Tornadijo M.E., Fresno-Baro J.M. (2019). Bioactive Components and Antioxidant and Antibacterial Activities of Different Varieties of Honey: A Screening Prior to Clinical Application. J. Agric. Food Chem..

[16] Aazza S., Lyoussi B., Antunes D., Miguel M.G. (2013). Physicochemical characterization and antioxidant activity of commercial portuguese honeys. J. Food Sci..

[17] Alvarez-Suarez J.M., Giampieri F., Brenciani A., Mazzoni L., Gasparrini M., González-Paramás A.M., Santos-Buelga C., Morroni G., Simoni S., Forbes-Hernández T.Y. (2018). Apis mellifera vs. Melipona beecheii Cuban polifloral honeys: A comparison based on their physicochemical parameters, chemical composition and biological properties. LWT Food Sci. Technol..

[18] Perna A., Intaglietta I., Simonetti A., Gambacorta E. (2013). A comparative study on phenolic profile, vitamin C content and antioxidant activity of Italian honeys of different botanical origin. Int. J. Food Sci. Technol..

[19] Kishore R.K., Halim A.S., Syazana M.N., Sirajudeen K.N.S. (2011). Tualang honey has higher phenolic content and greater radical scavenging activity compared with other honey sources. Nutr. Res..

[20] Kishore R.K., Halim A.S., Syazana M.N., Sirajudeen K.N.S. (2018). Malaysian stingless bee and Tualang honeys: A comparative characterization of total antioxidant capacity and phenolic profile using liquid chromatography-mass spectrometry. LWT Food Sci. Technol..

[21] Kaygusuz H., Tezcan F., Erim F.B., Yildiz O., Sahin H., Can Z., Kolayli S. (2016). Characterization of Anatolian honeys based on minerals, bioactive components and principal component analysis. LWT Food Sci. Technol...

[22] Ferreira I.C., Aires E., Barreira J.C., Estevinho L.M. (2009). Antioxidant activity of Portuguese honey samples: Different contributions of the entire honey and phenolic extract. Food Chem..

[23] Kesić A., Mazalović M., Crnkić A., Ćatović B., Hadžidedic Š., Dragošević G. (2009). The influence of L-ascorbic acid content on total antioxidant activity of bee-honey. Eur. J. Sci. Res..

[24] Nagai T., Kai N., Tanoue Y., Suzuki N. (2018). Chemical properties of commercially available honey species and the functional properties of caramelization and Maillard reaction products derived from these honey species. J. Food Sci. Technol..

[25] Dumbrava D.G., Bordean D.M., Raba D.N., Druga M., Moldovan C., Popa M.V. Antioxidant Properties and Other Physicochemical Characteristics of Some Honey Varieties from West Romanian Area. Proceedings of the 13th International Multidisciplinary Scientific GeoConference of Modern Management of Mine Producing, Geology and Environmental Protection (SGEM 2013).

[26] Islam A., Khalil I., Islam N., Moniruzzaman M., Mottalib A., Sulaiman S.A., Gan S.H. (2012). Physicochemical and antioxidant properties of Bangladeshi honeys stored for more than one year. BMC Complement. Altern. Med..

[27] Karabagias I.K., Maia M., Karabagias V.K., Gatzias I., Badeka A.V. (2018). Characterization of eucalyptus, chestnut and heather honeys from Portugal using multi-parameter analysis and chemo-calculus. Foods.

[28] Karabagias I.K., Karabournioti S., Karabagias V.K., Badeka A.V. (2020). Palynological, physico-chemical and bioactivity parameters determination, of a less common Greek honeydew honey: ‘dryomelo’. Food Control..

[29] Agus A., Agussalim N., Umami N., Budisatria I.G.S. (2019). Evaluation of antioxidant activity, phenolic, flavonoid and Vitamin C content of several honeys produced by the Indonesian stingless bee: Tetragonula laeviceps. Livest. Res. Rural Dev..

[30] Bundit T., Anothai T., Pattaramart P., Roongpet T., Chuleeporn S. (2016). Comparison of antioxidant contents of Thai Honeys to Manuka Honey. Malays. J. Nutr..

[31] Cosme P., Rodríguez A.B., Espino J., Garrido M. (2020). Plant phenolics: Bioavailability as a key determinant of their potential health-promoting applications. Antioxidants.

[32] Cianciosi D., Forbes-Hernández T.Y., Afrin S., Gasparrini M., Reboredo-Rodriguez P., Manna P.P., Zhang J., Bravo Lamas L., Martínez Flórez S., Agudo Toyos P. (2018). Phenolic compounds in honey and their associated health benefits: A review. Molecules.

[33] Khoddami A., Wilkes M.A., Roberts T.H. (2013). Techniques for analysis of plant phenolic compounds. Molecules.

[34] Lianda R.L., Sant’Ana L.D.O., Echevarria A., Castro R.N. (2012). Antioxidant activity and phenolic composition of Brazilian honeys and their extracts. J. Braz. Chem. Soc..

[35] Can Z., Yildiz O., Sahin H., Turumtay E.A., Silici S., Kolayli S. (2015). An investigation of Turkish honeys: Their physico-chemical properties, antioxidant capacities and phenolic profiles. Food Chem..

[36] do Nascimento K.S., Sattler J.A.G., Macedo L.F.L., González C.V.S., de Melo I.L.P., da Silva Araújo E., Granato D., Sattler A., de Almeida-Muradian L.B. (2017). Phenolic compounds, antioxidant capacity and physicochemical properties of Brazilian Apis mellifera honeys. LWT Food Sci. Technol..

[37] Khalil M.I., Alam N., Moniruzzaman M., Sulaiman S.A., Gan S.H. (2011). Phenolic Acid Composition and Antioxidant Properties of Malaysian Honeys. J. Food Sci..

[38] Ibrahimi H., Hajdari A. (2020). Phenolic and flavonoid content, and antioxidant activity of honey from Kosovo. J. Apic. Res..

[39] Šarić G., Marković K., Major N., Krpan M., Uršulin-Trstenjak N., Hruškar M., Vahčić N. (2012). Changes of antioxidant activity and phenolic content in acacia and multifloral honey during storage. Food Technol. Biotechnol..

[40] Neilson A.P., Goodrich K.M., Ferruzzi M.G. (2017). Bioavailability and metabolism of bioactive compounds from foods. Nutrition in the Prevention and Treatment of Disease.

[41] Kumar S., Pandey A.K. (2013). Chemistry and Biological Activities of Flavonoids: An Overview. Sci. World J..

[42] Kumar V., Suman U., Yadav S.K. (2018). Flavonoid secondary metabolite: Biosynthesis and role in growth and development in plants. Recent Trends Tech. Plant. Metab. Eng..

[43] Panche A.N., Diwan A.D., Chandra S.R. (2016). Flavonoids: An overview. J. Nutr. Sci..

[44] Sousa J.M., De Souza E.L., Marques G., Meireles B., de Magalhães Cordeiro Â.T., Gullón B., Pintado M.M., Magnani M. (2016). Polyphenolic profile and antioxidant and antibacterial activities of monofloral honeys produced by Meliponini in the Brazilian semiarid region. Food Res. Int..

[45] Al-Farsi M., Al-Amri A., Al-Hadhrami A., Al-Belushi S. (2018). Color, flavonoids, phenolics and antioxidants of Omani honey. Heliyon..

[46] Mabry T.J., Markham K.R., Thomas M.B. (1970). The Ultraviolet Spectra of Flavones and Flavonols. The Systematic Identification of Flavonoids.

[47] Dutra R.P., Abreu B.V.D.B., Cunha M.S., Batista M.C.A., Torres L.M.B., Nascimento F.R.F., Ribeiro M.N.S., Guerra R.N.M. (2014). Phenolic acids, hydrolyzable tannins, and antioxidant activity of geopropolis from the stingless bee melipona fasciculata smith. J. Agric. Food Chem..

